# Systematic Development of the YouRAction program, a computer-tailored Physical Activity promotion intervention for Dutch adolescents, targeting personal motivations and environmental opportunities

**DOI:** 10.1186/1471-2458-10-474

**Published:** 2010-08-11

**Authors:** Richard G Prins, Pepijn van Empelen, Marielle A Beenackers, Johannes Brug, Anke Oenema

**Affiliations:** 1 Department of Public Health, Erasmus University Medical Center, Rotterdam, the Netherlands; 2 EMGO Institute for Health and Care Research and the Department of Epidemiology & Biostatistics, VU University Medical Center, Amsterdam, the Netherlands

## Abstract

**Background:**

Increasing physical activity (PA) among adolescents is an important health promotion goal. PA has numerous positive health effects, but the majority of Dutch adolescents do not meet PA requirements. The present paper describes the systematic development of a theory-based computer-tailored intervention, YouRAction, which targets individual and environmental factors determining PA among adolescents.

**Design:**

The intervention development was guided by the Intervention Mapping protocol, in order to define clear program objectives, theoretical methods and practical strategies, ensure systematic program planning and pilot-testing, and anticipate on implementation and evaluation. Two versions of YouRAction were developed: one that targets individual determinants and an extended version that also provides feedback on opportunities to be active in the neighbourhood. Key determinants that were targeted included: knowledge and awareness, attitudes, self-efficacy and subjective norms. The extended version also addressed perceived availability of neighbourhood PA facilities. Both versions aimed to increase levels of moderate-to-vigorous PA among adolescents. The intervention structure was based on self-regulation theory, comprising of five steps in the process of successful goal pursuit. Monitoring of PA behaviour and behavioural and normative feedback were used to increase awareness of PA behaviour; motivation was enhanced by targeting self-efficacy and attitudes, by means of various interactive strategies, such as web movies; the perceived environment was targeted by visualizing opportunities to be active in an interactive geographical map of the home environment; in the goal setting phase, the adolescents were guided in setting a goal and developing an action plan to achieve this goal; in the phase of active goal pursuit adolescents try to achieve their goal and in the evaluation phase the achievements are evaluated. Based on the results of the evaluation adolescents could revise their goal or choose another behaviour to focus on. The intervention is delivered in a classroom setting in three lessons. YouRAction will be evaluated in a cluster-randomized trial, with classes as unit of randomization. Evaluation will focus on PA outcomes, cognitive mediators/moderators and process measures.

**Discussion:**

The planned development of YouRAction resulted in two computer-tailored interventions aimed at the promotion of PA in a Dutch secondary school setting.

**Trial registration:**

NTR1923

## Background

Sufficient physical activity (PA) contributes to the prevention of a range of conditions such as overweight and obesity, cardiovascular diseases, cancers, depression and low bone mineral density [1,2] and is associated with improved quality of life [3,4]. However, physical activity levels are low in most Western countries, across all phases in the life course, including adolescence. The most recent Dutch PA recommendations are that adolescents should engage in moderate to vigorous PA (MVPA) for at least one hour per day to promote cardiovascular and general health, and to engage at least twice a week in vigorous PA to help enhance and maintain physical fitness, including flexibility, muscular strength and bone health [5]. In the Netherlands approximately one quarter of the adolescents comply with the MVPA norm [6]. These figures demonstrate that there is a need for physical activity promotion interventions for adolescents and that large numbers of adolescents need to be reached with such interventions. Since there are not many PA promotion interventions available that meet these requirements, we developed a new and innovative e-health promotion intervention.

A planned approach to intervention development is likely to increase the effectiveness of interventions [7], given that it assures an optimal fit with the most important determinants of the targeted behaviour, inclusion of behaviour change strategies that fit with those determinants, and a delivery format that is appealing and suitable for the target population [8]. E-health promotion interventions using a web based delivery format may have specific advantages; (1) the format may be appealing for adolescents and (2) it can be used to tailor interventions to unique characteristics of a person [9,10], which is likely to increase intervention attractiveness, personal relevance and effectiveness. Classically, most tailored interventions focused on cognitive individual determinants of health behaviours such as awareness, attitudes and self-efficacy. Socio-ecological models, however, also suggest an influence of environmental factors, on behaviour, including factors in the physical environment [11,12]. To take these environmental factors into account we incorporated tailored feedback on the opportunities to be physically active in the neighbourhood in which adolescents live in the intervention, which is a novelty in the field of tailoring research.

To evaluate the additional effect of this environmental information, two versions of the computer-tailored intervention were developed. Both interventions are identical, and provide personalised feedback on individual determinants and change processes, whereas the extended version in addition provides feedback on the availability of PA opportunities in the home environment.

In the first part of this paper we describe the results of the systematic development of the intervention following the intervention mapping (IM) protocol for goal directed, theory and evidence based development of interventions. In the second part we describe the evaluation protocol.

## Methods/Design

### Intervention development

**Figure 1:**
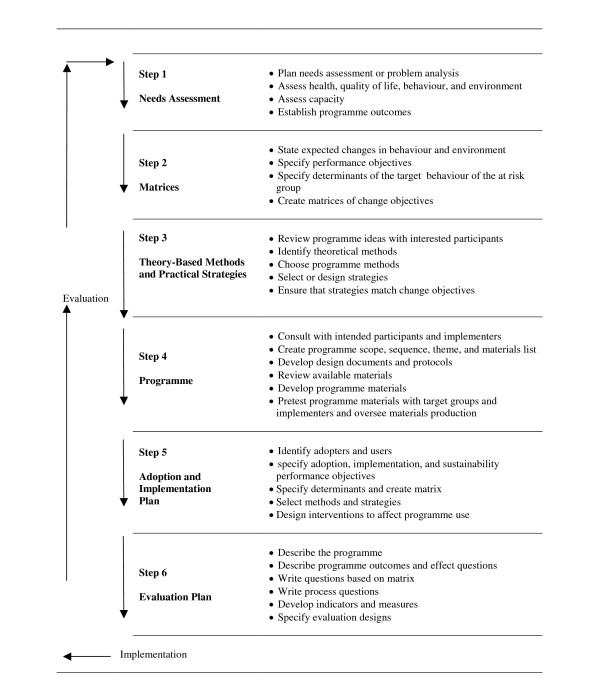
**Intervention Mapping protocol**.

The IM protocol describes six steps, in the process towards development of a theory-driven and evidence based intervention [8] (Figure 1). IM has successfully been applied in the development of interventions on promotion of a wide range of health behaviours/health states, including the prevention of overweight and obesity in adults [13] and adolescents [14,15] and promotion of physical activity in adults [16]. Below the results of each step in IM, which formed the basis for the two versions of the intervention, are described.

#### Step 1. Needs assessment

Carrying out a needs assessment is the first step in intervention development. In a needs assessment the discrepancy between the current situation and the desired situation in a given group of people is studied. Key points of the information generated in the needs assessment have already been described in the introduction section. To summarize, a sufficient level of MVPA is an important determinant for various diseases and health outcomes. MVPA may consist of activities such as brisk walking or cycling, leisure time physical activity (such as active ball games or dancing) or (organized) sports. However, a majority of the adolescents living in the Netherlands do not meet the Dutch MVPA guideline. Levels of MVPA in adolescents vary according to socio-economic status, gender and ethnicity, with in general lower levels of PA among lower educated groups [17,18] , girls [17-20] and ethnic minorities [18,20].

Based on the results of the needs assessment, we decided that the aim of this intervention was to achieve that an additional 10% of the adolescents meet the PA guidelines for moderate intensity physical activity for at least 60 minutes per day, at 6 months after exposure to the intervention." We deliberately stated this goal in such general terms, because it can be reached by engaging in a range of different PA sub-behaviours such as active transport, leisure time physical activity or participation in organized sports, recognizing the complexity and diversity of PA behaviour.

#### Step 2. Matrices of performance and change objectives

Once the overall health behavioural aim of the intervention was set, we defined what adolescents actually would need to do to achieve the overall intervention goal. Basically, adolescents need to comply with the MVPA guideline. Therefore, the intervention was developed to increase MVPA among adolescents in such a way, that adolescents engage in moderate intensity PA for 60 minutes per day on 7 days per week. This can be achieved by engaging in the previously defined PA sub-behaviours.

Behaviours, especially complex behaviours - such as MVPA - can mostly not be changed directly, but have to be broken down in smaller, specific actions to be taken, for instance "identifying sub-behaviour which can be improved" or "setting challenging goals". These more specific actions required in the process towards overall PA behaviour change are called, "performance objectives" in IM. Performance objectives were stated for the three identified sub-behaviours, sports, active transport and leisure time physical activity. All performance objectives in our intervention were based on theories of self-regulation. Self-regulation has been defined as "*a goal-guidance process, occurring in iterative phases, that requires the self-reflective implementation of various change and maintenance mechanisms that are aimed at task- and time-specific outcomes*." [21]. We distinguished five phases toward successful goal pursuit: a monitoring phase, a motivational phase, a goal setting phase, a phase of active goal pursuit and an evaluation phase (Table 1). In each phase other actions (i.e. performance objectives (PO)) are expected from the adolescents (Table 1). For instance, in the monitoring phase adolescents monitor their current behaviour (PO1a, Table 1), in the motivational phase, adolescents decide to increase the selected PA activity (PO2c, Table 1), in the goal setting phase adolescents set a goal to increase the selected PA activity (PO3a, Table 1) and in the phase of active goal pursuit, adolescents engage in activities to achieve this goal (PO4a, Table 1). These proceedings are monitored in the last phase, in which adolescents evaluate their achievements in terms of goal attainment (PO5a, Table 1). Based on the outcome of the evaluation adolescents can decide to maintain their goal, (PO5c, Table 1) or, in case of failure to attain their goal, to choose new strategies to reach their goal or select a new goal (PO 5b, Table 1). Finally, adolescents have to set a long-term goal and create a plan to achieve this (PO5c, Table 1). Because the performance objectives were based on self-regulation theory, they also serve as a general outline of the intervention. A complete list of performance objectives is displayed in Table 1.

**Table 1:** Performance objectives per "self-regulation" phase

Self-regulation phase	Performance objectives
Monitoring	Adolescents monitor current state of PA (PO1a)
	Adolescents identify sub-behaviours which can be improved (PO1b)

Motivational phase	Adolescents decide to increase PA (PO2a)
	Adolescents select a PA activity that fits with their personal preference (PO2b)
	Adolescents are decide to increase the PA activity of personal preference (PO2c)

Goal setting	Adolescents set challenging, but feasible PA goal (PO3a)
	Adolescents create an action plan to achieve PA goal (PO3b)

Active goal pursuit	Adolescents engage in activities to accomplish PA goal (PO4a)

Evaluation	Adolescents monitor their achievements (PO5a)
	Adolescents decide, based on their achievements, to proceed pursuing their goal or state a new goal (PO5b)
	Adolescents make a long-term planning (PO5c)

PO = performance objective

Another main task in the phase of developing specific intervention objectives is that important and changeable determinants of the target behaviours and sub-behaviours are identified. These important and modifiable determinants of physical activity in adolescents were selected based on a review of the literature and identification of additional constructs from theories. For the identification of additional constructs we consulted theories that are predominantly used in the field of PA and other health related behaviours, including the Theory of Planned Behaviour (TPB)[22], the Social Cognitive Theory (SCT)[12], the EnRG Framework[11] and the Precaution Adaptation Process Model (PAPM)[23]. Attitude [24-27], subjective norm [24-26,28], self-efficacy/perceived behavioural control [24-26,29-33] and intention were identified as important determinants of PA behaviour among adolescents in the scientific literature. Derived from theory and studies among adolescents, we identified awareness of one's own behaviour as an important additional determinant for complex behaviours such as physical activity [23].

In addition, one of the recent developments in the field physical activity is the interest in socio-ecological models to explain health behaviour. Some of these models propose an effect of the physical, socio-cultural, political and economic environment on PA directly or indirectly via cognitive variables [11,12]. Research to date indicates that there is often a mismatch between the objective and perceived environment [19,34]. Frequently, people underestimate the existence of physical activity opportunities in their environment. Adolescents may not be aware of all the opportunities for PA in their home neighbourhood. Thus, we identified perceptions of the environment as another additional determinant to be addressed in the intervention.

As a final task in this step of IM, the selected determinants and performance objectives were integrated in matrices to define change objectives. Change objectives define what the participants need to learn or do, in order to achieve the performance objectives. For instance, in order to achieve the performance objective "Adolescents decide to increase sports participation", an important determinant is self-efficacy. One of the change objectives in terms of self-efficacy is "Adolescents identify difficult situations which may prevent them from participating in sports". In the intervention development process, the change objectives were used to select appropriate behaviour change methods and to produce the right content of the intervention. A selection of all the change objectives is shown in Table 2. For all three sub-behaviours, separate matrices of change objectives were created.

**Table 2:** Matrix of change objectives: an example for selected determinants of the sub-behaviour sports

Performance objective	Attitude	Subjective norm	Self-efficacy/Perceived behavioural control	Awareness	Perceived environment
*Adolescents decide to increase sports participation (PO2A)*	Adolescents feel sports is enjoyable	Adolescents comply with the norm to engage in sports on a regular base	Adolescents identify difficult situations which may prevent them from participating in sports	Adolescents monitor their current sports behaviour	Adolescents know where facilities to engage in the sports they want to are in their neighbourhood
	Adolescents see the health benefits of engaging in sports		Adolescents are confident that they can cope with barriers	Adolescents compare their sports behaviour with the fitness norm	
				Adolescents compare their sports behaviour with peers	

#### Step 3. Methods and strategies in a general outline

In the next step of Intervention Mapping, appropriate methods, derived from literature, to modify the determinants were selected for each specific change objective. These theoretical methods were translated into practical strategies that can be delivered in the intervention, by taking their parameters for use (i.e., important criteria that need to be met for a strategy to be effective [8]) into account. In the description of the methods we used the descriptions as suggested by Abraham and Michie in their taxonomy of behaviour change techniques [35]. Since the intervention is computer-tailored, all methodologies were tailored to the adolescents needs. An overview of the main methods and strategies applied in the intervention and the theories from which they were derived is shown in Table 3. The self-regulation phases were the basis for the outline of the program. Program development was part of the next step of Intervention Mapping (step 4), but because of comprehensibility and readability of this paper we decided to integrate the description of the program outline in the methods and strategies section.

**Table 3:** Most used methods in YouRAction, their parameters for use and examples of strategies

Determinant	Technique/Method (theory)	Parameters for use	Strategy
Perceived environment	Facilitation		Providing sports clubs on an interactive map, tailored around the adolescents' home;
	
	Facilitation		Providing walking and cycling routes
			Providing visual representation of walking and cycling possibilities in neighbourhood
	
	Active learning		Adding places to be active on maps

Knowledge	Provide information about behaviour-health link (PAPM)	Adding helpful information	Adolescents first fill in a practice quiz, which gives feedback. Subsequently they fill in a quiz which will be marked.
	
	Advance organizers	Schematic representations of the content or guided to what is to be learned	Schematic on levels of PA, with colours

Attitude	Modeling	Reinforcement of the model	Web movie with models who decided to be active. Models smile and name the pro's they experienced of activity.
	
	Provide information on consequences (TPB, SCT)	Investigation of the current beliefs of the individual before choosing the belief on which to intervene	Feedback based on pros and cons of specific behaviour

Awareness	Provide information about behaviour-health link (PAPM)	Messages should be presented as individual, undeniable, on the same dimension, congruent with actual risk, and cumulative rather than for one occasion messages presented with qualitative and quantitative examples	Feedback based on PA questionnaire on overall PA and sub-behaviours. Feedback consists of individual and normative feedback in text and graphs.

Subjective norm	Building skills for resistance to social pressure (SCT)	Skill building for refusal skills; commitment to earlier intention; relating intended behaviour to values; psychological inoculation against pressure	Simulation of role-plays by making use of interactive web movies.

Self-efficacy/PBC	Prompt barrier identification (SCT)	Choosing barriers which are important to adolescents	List of barriers in which adolescents can select barriers
	
	Model or demonstrate the behaviour (SCT)/Set graded tasks (SCT)	Subskill demonstration, instruction and enactment with feedback	Web movies and comics in which in steps a solution to a problem is presented.
	
	Relapse prevention (relapse prevention therapy)	Identification of high-risk situations and practice of coping response	Selection of barriers to be active in a predefined list. Followed by guided practice to cope with barrier and a homework assignment to practice this

Planning	Prompt specific goal setting (CT)	Commitment to the goal; goals that are difficult but achievable	Setting goals using a step-by-step application.
	
	Relapse prevention (relapse prevention therapy)	Identification of high-risk situations and practice of coping response	Selection of barriers. Followed by form to fill in strategy to overcome barrier.
	
	Prompt practice (OC)	Encourage adolescents to carry out their plan	Practice in achieving goals for one week. Daily feedback on achievement and barriers that occurred during that day

Relapse prevention	Prompt self-monitoring of behaviour (CT)/Provide feedback on performance (CT)	Can use feedback and confrontation; however raising awareness must be quickly followed by increase in problem-solving ability and self-efficacy	After goal setting, daily monitoring of behaviour with feedback on achievement.
			Daily feedback on progress to achieve goal. If adolescents fail, feedback makes them aware that behavioural change goes hand in hand with occasional failure.
	
	Agree on behavioural contract	Creating of behavioural contract which will be signed by adolescent and teacher	Step-by-step creation of behavioural contract, containing goal, plan to achieve this goal and reward when achieving this goal. This control can be printed to be signed by adolescent and teacher
	
	Prompt review of behavioural goals (CT)	First guided monitoring, then self-monitoring. Feedback based on this self-monitoring in terms of behavioural goal.	Guided monitoring after second lesson; adolescents monitor their achievements. After third lesson they will be stimulated to this by their own in their diary. Based on these results resetting behavioural goals or progress with current goal.

**Figure 2:**
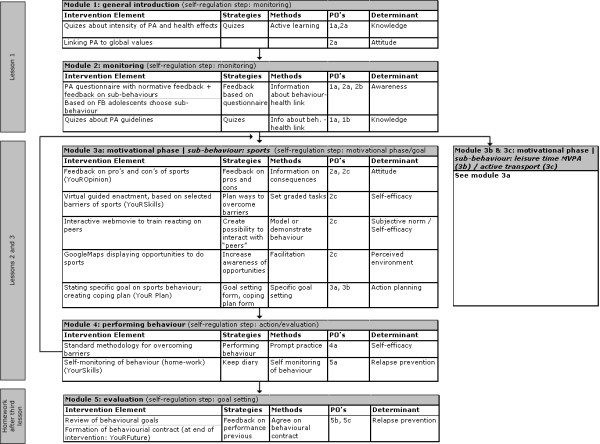
**Schematic Presentation of Intervention**.

TPB = theory of planned behaviour; SCT = social-cognitive theory; CT = control theory, PAPM = precaution adoption process model;

The program consists of three lessons of approximately 35 minutes, with homework assignments to be completed between the lessons. All lessons consist of one or more sections in which specific determinants were targeted. These sections are based on the general theme of the intervention: "YouR". The name of the intervention, YouRAction, is an acronym of Youths of Rotterdam in Action. Examples of section names include YouROpinion (targeting attitudes) and YouRSkills (targeting self-efficacy). A general outline of the intervention is shown in Figure 2.

##### First time use

The program is introduced by the teacher, after which the adolescents sign in to the program. They can than choose their personal guide, to increase attention for the messages throughout the program. Adolescents can choose between two "personal guides": Armin and Anna. This guide appears in movies, comics and throughout the program, mostly in an animated format.

##### Monitoring phase

In the monitoring phase of self-regulation, adolescents are first made familiar with the terminology used throughout the intervention and learn about various levels of PA. Knowledge is targeted in the section "YouRKnowledge", by making use of the methodology of active learning. In practice, this means that interactive quizzes and feedback are used to test, and increase, their knowledge on concepts of PA and terminology used throughout the intervention. Before the actual monitoring starts, attitudes towards PA in general are addressed by linking physical activity with general values. Adolescents can pick two important values from a predefined list (e.g. "Nature and the environment important for me") on which feedback is provided how physical activity fits with that value.

The section YouRBehaviour, deals with monitoring and awareness of personal PA levels. Personal feedback on behaviour and normative feedback are the main methods used to increase awareness of PA levels. A detailed questionnaire on PA sub-behaviours is used to monitor current PA status and is used to generate individualized tailored feedback on PA level and how this relates to the PA norms and PA level of peers. This feedback is shown in bar charts, with a textual explanation. Subsequently, more detailed feedback on performance in the three PA sub-behaviours (i.e. sports, active transport and leisure time physical activity) is provided, to indicate in which sub-behaviours improvements can be made.

After completing the YouRBehaviour section, adolescents proceed to YouRMove, in which they have to make a decision whether or not to improve their PA-level and on which sub-behaviour they would like to focus, i.e., active transport, leisure time activity or sports (performance objective 1b, Table 2). This decision is made at the end of the first lesson.

##### Motivational phase

The second and third lesson both start with further motivating the adolescents for making changes in the chosen behaviour (Figure 2). Attitude, self-efficacy, subjective norm and the perceived environment were identified as important determinants to be addressed in this phase.

In YouROpinion, attitude is addressed by means of two methods: modelling/demonstrating behaviour and providing information on consequences of the behaviour. This means that, in the program, attitude is targeted by means of a web movie in which models tell about the positive sides of the chosen behaviour, emphasizing the fun and positive feelings associated with this behaviour (method: model or demonstrate the behaviour/provide information on consequences). Subsequently, adolescents select their own pros and cons of the sub-behaviour. For instance, adolescents may perceive as pros for sports "I will have a relaxed feeling after playing sports", "Sports is healthy" or "I can meet (new) friends". Whereas, cons of sports may include "Sports makes me feel sweaty", "Sports causes muscle soreness" or "Sports is too time consuming". When this balance is positive, the advantages of the behaviour are reinforced. In the case that this balance is negative, the cons are hypothetically discussed by a virtual panel of adolescents, aimed to shift the perspective of the adolescent (method: provide information on consequences). The initiation of these texts is from the initial perspective of the adolescent, meaning that the adolescent of the panel initially experienced the disadvantage too, but is now convinced that this perceived disadvantage is not a real disadvantage.

The section in which self-efficacy is the central theme is called YouRSkills. Self-efficacy is targeted by three main methods: prompting identification of barriers, model or demonstrate behaviour and setting graded tasks [8,35]. First adolescents select barriers which may prevent them from performing the selected sub-behaviour (method: prompt barrier identification). For example, bad weather, time, not knowing how to get to places and travelling alone are barriers that may prevent adolescents from travelling by active transport. The program contains interactive assignments, web movies or comics to overcome the selected barriers. In all assignments, problem solving strategies are incorporated: 1) what is the problem, 2) what can you do about it, 3) try it! Examples of these interactive assignments include interactive web movies to learn how to react on peer pressure (methods: model or demonstrate the behaviour/set graded tasks). Or, when bad weather is a barrier, adolescents are taught how to figure out what the weather forecasts are for the rest of the day - using web based weather forecasts and TV based weather forecasts. Finally, they plan coping responses on what to do in the case of bad weather, or any of the other barriers identified.

Feedback on the availability of PA facilities was used as the method to target self-efficacy and perceptions of the physical environment, in the section YouR'Hood. This was done by showing which PA facilities are available in an adolescent's neighbourhood environment using GoogleMaps. Environmental feedback was provided in various ways, depending on the PA sub-behaviour of interest. One of the applications is that adolescents can point out on the map where they can do the activity of their choice, e.g., where they can play tennis; this is applied to promote sports participation and leisure time physical activity. Another application is that adolescents can create routes to different places; this is applied to promote active transport. In the third strategy applied, all facilities in the neighbourhood are displayed on the map to give adolescents an overview of what is available in their neighbourhood; this is used to promote sports participation (i.e. sports facilities and sports clubs), leisure time physical activity and active transport (i.e. parks). Facilities can be selected and additional information about the location pops up, such as of contact details and web addresses of sports clubs, or information on quality of a park when a park was selected. Examples of these strategies can be viewed on several demo movies (Additional file 1, Additional file 2). All maps are tailored to the home address, meaning that the map is centred on the home address. This means that an adolescent will immediately see which opportunities to be active are present in his or her neighbourhood.

##### Goal setting phase

The section "YouRGoal" is designed to achieve the performance objective "Adolescents set challenging, but feasible PA goal". Important prerequisites for goal setting are that the goals are challenging, but feasible [36]. To make sure this is the case, we used a closed ended format for setting a goal. First a goal is set by selecting one goal out of four pre-defined goals, such as "In the next week I am going to cycle to school four times". If the goal an adolescent wants to set is not in the predefined list, there is an option to set a goal using a step-by-step guide [36]. In that case, adolescents first choose what to do, for instance "Playing basketball". Then adolescents select how many times they want to do this in the next week and on which days they plan to do this.

Once the goal is set, adolescents enter "YouRPlan" and plan their activities in a calendar for seven consecutive days (method: prompt specific goal setting). They can click on the activity and a pop-up occurs in which they can state with whom and where they plan to do the activity (i.e. implementation intentions [37] are created). In the same pop-up window adolescents can plan coping responses to selected barriers. Such a coping response may look like: "When it rains, I will take my rain gear with me." The adolescent can choose to have the complete plan sent to his or her e-mail address, or to print it directly from the web page.

##### Phase of active goal pursuit

In the phase of active goal pursuit, adolescents work on executing their plans to achieve their goals. Every day, during one week after the second lesson, the adolescents log in to the website and fill in what activities they did, which difficult situations occurred and whether they managed to overcome these barriers (method: prompt self-monitoring of behaviour). If they did not manage to overcome these barriers, the adolescents enter a problem solving module, in which failure when trying to do something new is attributed to something that may occur occasionally. Subsequently, they are stimulated to try to think of a way to overcome this barrier the next time and write it down in the program (method: Relapse prevention). In the third lesson this part is slightly modified, as adolescents are stimulated to monitor their behaviour on their own; for instance by making use of their (school) agenda.

##### Evaluation phase

At the beginning of the third lesson, adolescents receive feedback on their performance during the past week, in YouRWeek (method: feedback on performance). Based on this feedback adolescents can choose either to 1) try to reach the same goal again 2) state a new goal on the same sub-behaviour 3) work on another sub-behaviour. Subsequently adolescents enter the motivational phase again, as can be seen in the feedback loop from Module 4 to Module 3 (Figure 2) (method: prompt review of behavioural goals).

In YouRFuture, which is homework after the third lesson and therewith the very last part of the intervention, attention is paid to long term goals, by means of the methods prompt specific goal setting, relapse prevention, prompt self-monitoring and agree on behavioural contract. Adolescents set long-term (half year) goals on moderate-to-vigorous physical activity and sports, using pre-defined goals. Subsequently, they create an action plan and explicitly state when they monitor their behaviour and which incentive they give themselves, when they are doing well in reaching their goal. These plans are synthesized to a behavioural contract. This contract will be signed by themselves and their teacher.

#### Step 4. Producing program components and materials

The general program outline has already been described in the previous section. Therefore the focus of this section is on the very last part of intervention development: the production of the final materials and pretesting of the developed materials.

##### Technical development

YouRAction was developed in Tailorbuilder software. This software enables intervention developers to create their own web based computer tailored interventions. In order to be able to provide environmental feedback (i.e. show geographic information), extensions to the Tailorbuilder software had to be made. Building a system able to show geographic information from scratch would be very expensive. Instead, the Application Programming Interface (API) of GoogleMaps, provided by Google was used. This proved to be a reliable and efficient way to extent our Tailoring program.

For incorporation of environmental information in our system, the same tailoring principle was used as in other tailoring. A score on a tailoring variable is used to select the relevant feedback messages from a computer database, which is subsequently shown on the screen. In other words, environmental feedback had to be tailored to the relevant tailoring variable: the postal code of the home address. The tailoring algorithms defined that all the PA facilities in a 5000 meter radius around the home address had to be selected from the message library and plotted on a map. Thus the geographic locations of opportunities to be active formed the tailored environmental feedback. The environmental information in the database/message library was retrieved from municipal websites and parks were observed by trained observers using an adapted and translated version of the POST audit Tool (POST) [38].

##### Pre-test and formative evaluation

The intervention was developed in collaboration with adolescents, teachers and behaviour change experts, who participated in a linkage group. This linkage group met twice in an official meeting and was contacted on a question-answer base by mail or phone. The adolescents and teachers were approached in several phases of the development process, to give feedback on specific intervention elements, such as the use of GoogleMaps, the use and placement of images and movies and the use of an activity planner.

**Figure 3:**
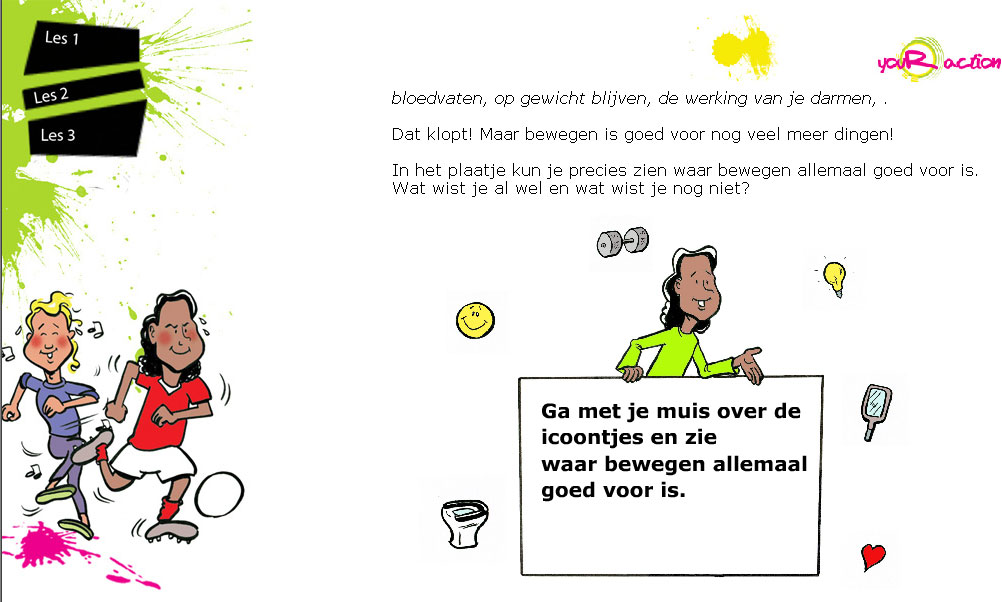
**Visual design of the intervention**.

Materials were developed and pre-tested by members of the linkage group, adolescents and teachers. Adolescents were consulted to choose one of three intervention names and one of three logos. The web movies were shot in cooperation with adolescents, who played a role in these movies. Their input was also taken into account to shape the final script of the movies. The graphical design of the final website was developed by adolescent students of a school for graphic design (Figure 3). The final intervention was pre-tested by adolescents and teachers in a classroom setting. This gave us information about usability of the intervention in a classroom setting. During this pre-test some technical problems (speed problems and problems with signing in) occurred, which were solved.

We specifically focussed on pre-testing the incorporation of GoogleMaps in the tailoring software, since this was an innovative element. First, we started with observing the use of GoogleMaps in a classroom. Based on these observations some adaptations to the use of GoogleMaps were made, such as fine-tuning the radius in which facilities were shown. Secondly, we pre-tested the GoogleMaps application with the adaptations made in a classroom setting (N = 51 adolescents). Adolescents rated the incorporation of GoogleMaps positively. Most adolescents found that 1) the instructions were clear, 2) the map was easy to use, 3) it was fun to work with the maps and 4) the information was complete.

### Evaluation study

#### Interventions

The efficacy of the regular and extended version of the computer-tailored intervention will be evaluated against an Internet delivered intervention that provides generic information about physical activity and nutrition. All three interventions have the same graphical design and lay-out and they all consisted of three lessons. The lessons will be given by teachers who are provided with a teacher manual, as a guide for using the program.

#### Aim of evaluation study

The aim of the evaluation study is to evaluate achievement of the intervention goals that have been stated during the intervention development process. Furthermore, the aim is to identify whether changes in the outcome measures were mediated through changes in the underlying processes. Therefore, effects of the interventions on behavioural determinants, on PA behaviour and mediation of intervention effects by targeted determinants will be studied. The main outcome measures are compliance to the PA guidelines, time spent in PA and specific sub-behaviours and behavioural determinants. Additionally, the intervention will be evaluated on the process level on acceptability and implementation of the intervention.

The main research question to be answered is whether 10% more adolescents in the intervention groups, as compared to the control group, comply with the MVPA norm at six months post intervention.

#### Evaluation design

The YouRAction intervention will be evaluated in a three-armed cluster-randomized trial, with school classes as the unit of randomization, using computer aided block-randomization with a block size of 9. In each arm, 17 classes with on average 22 respondents per class will be needed to detect a 10% difference in compliance with the MVPA norm at six months post intervention (alpha = 0.05, power = 0.80, ICC = 0.02, %compliance with norm in control group = 15% (observed from baseline measurements of the ENDORSE study [18])). Measurements will take place at baseline (T0) (September/October 2009), one month post intervention (T1) (October-December 2009) and 6 months post intervention (T2) (April-June 2010) (Table 4). Questionnaires will be administered at each time point. A randomly selected subsample of 10% of the participants will be asked to wear ActiGraph GT3X accelerometers, on each time point, to objectively assess PA. Height and weight (to calculate Body Mass Index) and waist circumference will be assessed among a random selection le of 40% of the participants at T0 and T2. Random selection of those participating in the additional measurements will be done by using a random ranking system, in which the first ranked adolescents in each class were picked for the additional measurements. Random selection will be separate for the accelerometer usage and anthropometric measurements.

**Table 4:** Evaluation design

	T0	T1	T2
Timepoint	Baseline Sept-Oct 2009	1 month post intervention Oct-Dec 2009	5-6 months post intervention April-June 2010

Demographics	Gender, age, education, ethnic background		

Outcome measures	Self-reported PA Objectively measured PA Body Mass Index Waist circumference	Self-reported PA Objectively measured PA	Self-reported PA Objectively measured PA Body Mass Index Waist circumference

Determinants	All	All	All

Process evaluation	No	Yes	No

The Medical Ethics Committee of Erasmus University Medical Center provided a declaration of "no objection" for this study.

#### Measurement

Self-reported PA, will be measured by means of the AQuAA [39] and a sixty-minute screening measure for moderate to vigorous physical activity [40]. Secondary outcomes will include objectively assessed PA, by means of ActiGraph accelerometers and objectively assessed BMI and waist-circumference.

#### Determinants

Of all adolescents demographic information on age, gender, education and ethnic background will be collected. Adolescents will fill in a questionnaire at T0, T1 and T2 on cognitive determinants of MVPA, sports, walking, cycling and leisure time physical activity. The cognitive determinants assessed will include those determinants targeted in the intervention: awareness, attitudes, perceived behavioural control, subjective norm and intention, action planning and the perceived physical environment.

#### Process evaluation

Additionally at T1, a process evaluation will be conducted, to assess the intervention on acceptability, feasibility and implementation from the teacher's and adolescent's point of view. Teachers will be stimulated to fill in a log book on how much time each lesson took them in preparation and when they gave the lesson. Furthermore they will be stimulated to fill in an online questionnaire on their experiences with using the intervention.

In some classes research staff will observe intervention implementation and make notes of their observations. Furthermore server logs will be analyzed to detect how often en when the intervention was used.

#### Participants

Participants will be recruited in a two-step procedure. First, schools will be recruited by contacting health coordinators of schools. Second adolescents within the selected schools were recruited. School classes are eligible if they are first year classes, with the main language during teaching being Dutch. These schools should be located within the area of the Municipal Health Services Rotterdam and surroundings. All adolescents in the selected classes will be invited to participate. We will use a passive informed consent procedure, meaning that, after being fully informed about the study, its purpose and its procedures, the adolescent and/or their parents could refuse to participate.

## Discussion

The planning process that we described in this study has resulted in an extensive, novel, PA promotion intervention for adolescents that can be implemented in a school setting. Theory and evidence based development of interventions increases the likelihood of an intervention to be effective [7]. The present paper contributes to providing more insight into the systematic development of interventions and a more proper and detailed description of the behaviour change methods and strategies used, as has recently been called for [41].

Although this intervention is developed based on theory and evidence, its effectiveness still needs to be evaluated in an evaluation study. Moreover, there is little known about the efficacy of computer tailored interventions among adolescents. Evidence from our evaluation study will provide information on the efficacy of the intervention, potential mediating and moderating mechanisms and the added value of improving awareness of PA facilities in the environment. In addition, the study will provide insight in appreciation and implementation possibilities for the intervention in a school setting.

One of the novelties of this intervention is the incorporation of environmental feedback in a computer tailored intervention. This is a novelty for adolescents, but has successfully been applied in a tailored intervention in adults by van Stralen et al. [16]. Van Stralen et al. [16] send maps on opportunities to be active on paper to respondents of her intervention. This approach was found to be effective [42,43]. However, the Active+ intervention did not give instant, interactive feedback on opportunities to be active using tailored online maps. In our pretest it turned out that adolescents do have skills to work with GoogleMaps. Therefore we think that the incorporation of GoogleMaps to a computer-tailored intervention in adolescents is feasible and potentially effective.

Intervention Mapping was a useful tool in developing the YouRAction interventions, however, one of the main limitations was that the protocol is not clear in how to deal with complex behaviours like physical activity, in which various sub-behaviours can be chosen to achieve one overall goal. The aim of our intervention, increasing MVPA, could be reached by improving active transport, engagement in sports and leisure time activities - or in a combination of activities. However for each activity other determinants, change objectives and methods and strategies may be important. In order to be able to do so, we decided to create separate matrices of change objectives for each sub-behaviour, which enabled us to state specific change objectives for each sub-behaviour and select the appropriate methods and strategies to meet the change objectives. The technique of computer tailoring furthermore facilitated the development of a program consisting of such a flexible program. In fact, big parts of the interventions were tailored to the adolescents' selection of a PA sub-behaviour. However, creating programs which are too complex may need more tailoring algorithms, which may have negative impact on the speed of the program.

To conclude, the development of YouRAction based on the Intervention Mapping protocol resulted in two theory and evidence based computer-tailored interventions with innovative elements like the incorporation of GoogleMaps. An evaluation study has to provide insight into the efficacy of the basic and extended intervention and will provide evidence for the additional effect of the provision of environmental feedback for computer-tailored PA interventions. If the evaluation study proves that the interventions are effective, two well-developed interventions will become available for the promotion of PA among adolescents.

## Competing interests

The authors declare that they have no competing interests.

## Authors' contributions

All authors contributed to drafting the manuscript and approved the final version. RGP, AO and JB designed the intervention and the evaluation study. All authors contributed to the development of the intervention. All authors have read and approved the final manuscript.

## Pre-publication history

The pre-publication history for this paper can be accessed here:

http://www.biomedcentral.com/1471-2458/10/474/prepub

## Supplementary Material

Additional file 1 **Demo movie of an interactive map to explore the home neighbourhood**. This movie shows strategies incorporated in the YouRAction intervention to actively explore possibilities to be physically active in the home neighbourhood. Click here for file

Additional file 2 **Demo movie of an interactive map showing availability of parks**. This movie shows strategies incorporated in the YouRAction intervention to change perceptions of availability of parks in the neighbourhood by giving adolescents an overview of what is available. Click here for file
